# Effectiveness of full Pulpotomy compared with Root canal treatment in managing teeth with signs and symptOms indicative of irreversible pulpitis: a protocol for prospectiVE meta-analysis of individual participant data of linked randomised clinical trials (PROVE)

**DOI:** 10.1186/s13063-023-07836-6

**Published:** 2023-12-15

**Authors:** I. A. El Karim, H. F. Duncan, A. F. Fouad, N. A. Taha, V. Yu, S. Saber, V. Ballal, P. Chompu-inwai, H. M. A. Ahmed, B. P. F. A. Gomes, S. Abushouk, S. Cushley, C. O’Neill, M. Clarke

**Affiliations:** 1 Centre for Experimental Medicine, School of Medicine, Dentistry and Biomedical Sciences, Queen’s University Belfast, The Wellcome-Wolfson Building, 97 Lisburn Road, Belfast, BT9 7AE Northern Ireland, UK; 2 Division of Restorative Dentistry & Periodontology, Dublin Dental University Hospital, Trinity College Dublin, Lincoln Place, Dublin 2, Ireland; 3 School of Dentistry, The University of Alabama at Birmingham, Birmingham, AL USA; 4 Department of Conservative Dentistry, Faculty of Dentistry, Jordan University of Science and Technology, Irbid, Jordan; 5 Faculty of Dentistry, National University of Singapore, Singapore, Singapore; 6 Department of Endodontics, Faculty of Dentistry, The British University, Cairo, Egypt; 7 Department of Conservative Dentistry and Endodontics, Manipal College of Dental Sciences-ManipalManipal Academy of Higher Education, Manipal, India; 8 Division of Pediatric Dentistry, Department of Orthodontics and Pediatric Dentistry, School of Dentistry, Chiang Mai University, Chiang Mai, Thailand; 9 Department of Restorative Dentistry, Faculty of Dentistry, Universiti Malaya, Kuala Lumpur, Malaysia; 10 Department of Restorative Dentistry, Division of Endodontics, Piracicaba Dental School, State University of Campinas-UNICAMP, Av. Limeira Piracicaba, Areião, SP 90113414-903 Brazil; 11 Department of Oral Rehabilitation, Faculty of Dentistry, Khartoum University, Khartoum, Sudan; 12 Centre for Public Health, School of Medicine Dentistry and Biomedical Sciences, Queen’s University Belfast, Grosvenor Road, Belfast, BT12 6BJ N. Ireland UK

**Keywords:** Prospective meta-analysis, Pulpotomy, Root canal treatment, Irreversible pulpitis, Vital pulp treatment, Deep caries, Randomised trial

## Abstract

**Background:**

Full pulpotomy has been proposed as an alternative to root canal treatment in teeth with signs and symptoms indicative of irreversible pulpitis (IRP), but the evidence is limited, relying on underpowered studies with a high risk of bias. The aim of this study is to conduct a prospective meta-analysis (PMA) of individual participant data of a series of individual randomised trials to provide robust evidence on the clinical and cost-effectiveness of pulpotomy compared with root canal treatment.

**Methods:**

Individual participant data will be obtained from a series of randomised trials designed and conducted by a consortium of multi-national investigators with an interest in vital pulp treatment. These individualised trials will be conducted using a specified protocol, defined outcomes, and outcome measures. Ten parallel-group randomised trials currently being conducted in 10 countries will provide data from more than 500 participants. The primary outcome is a composite measure defined as (1) the absence of pain indicative of IRP, (2) the absence of signs and symptoms indicative of acute or chronic apical periodontitis, and (3) the absence of radiographic evidence of failure including radiolucency or resorption. Individual participant data will be obtained, assessed, and checked for quality by two independent reviewers prior to the PMA. Pooled estimates on treatment effects will be generated using a 2-stage meta-analysis approach. The first stage involves a standard regression analysis in each trial to produce aggregate data on treatment effect estimates followed by an inverse variance weighted meta-analysis to combine these aggregate data and produce summary statistics and forest plots. Cost-effectiveness analysis based on the composite outcome will be undertaken as a process evaluation to evaluate treatment fidelity and acceptability by patients and dentists.

**Results:**

The research question and trial protocol were developed and approved by investigators in all 10 sites. All sites use shared resources including study protocols, data collection forms, participant information leaflets, and consent forms in order to improve flow, consistency, and reproducibility. Each site obtained its own Institutional Review Board approval, and trials were registered in appropriate open access platforms. Patient recruitment has started in most sites, as of July 2023.

**Discussion:**

PMA offers a rigorous, flexible, and efficient methodology to answer this important research question and provide results with improved generalisability and external validity compared with traditional trials and retrospective meta-analyses. The results of this study will have implications for both the delivery of clinical practice and structured clinical guidelines’ development.

**Trial registration:**

PROSPERO CRD42023446809. Registered on 08 February 2023.

**Supplementary Information:**

The online version contains supplementary material available at 10.1186/s13063-023-07836-6.

## Background

Dental caries in permanent teeth is a common reason for adult patients to seek medical assistance for pain relief [1] and endodontic treatment [2]. If left untreated, caries will progress, inducing severe inflammation in the dental pulp, clinically diagnosed as irreversible pulpitis (IRP). The term “irreversible” was introduced based on older studies, indicating that conservative treatment of this inflamed pulp would not lead to healing but would result in pulp necrosis in most cases [3]. Therefore, IRP is traditionally treated by root canal treatment, which, although successful if carried out well [4, 5], is invasive, expensive, technically challenging, and time-consuming and risks the development of vertical root fracture [6, 7]. Root canal treatment in general dental practice is generally poorly performed, with recent UK data highlighting that a large proportion were technically inadequate, with a high prevalence of associated apical infection and disease [8]. The associated costs and need for the use of appropriate resources mean that this treatment will not be available for many patients in low- and middle-income countries where extraction is the only alternative for teeth with IRP [9].

Recent studies have shown that inflammation in teeth traditionally diagnosed with IRP is partial (i.e. limited to the coronal pulp tissue), with the absence of bacteria and inflammation in the radicular pulp tissue [10]. Moreover, a class of dental materials, namely hydraulic calcium silicate cements (HCSC), has been shown to exhibit unique mineralising, antimicrobial, and biocompatibility properties that have tremendously improved the outcomes of vital pulp therapy [11]. This has led to the introduction of new management strategies for IRP [12] aimed at preserving all or part of the dental pulp, including performing pulpotomies. Pulpotomy is a minimally invasive procedure whereby the inflamed/diseased pulp tissue is removed from the coronal pulp chamber of the tooth leaving healthy pulp tissue, which is dressed with a dental biomaterial that maintains vitality and promotes repair [11].

To date, several single-arm intervention studies (case series) have demonstrated a high success rate for full pulpotomy after carious pulpal exposure in patients with signs and symptoms indicative of IRP [13–15]. However, only a small number of studies have compared pulpotomy with root canal treatment [16–18]. A systematic review commissioned by the European Society of Endodontology (ESE) as part of the S3-level clinical guidelines for endodontic treatment found only two studies that met the inclusion criteria of symptomatic IRP [19]. Meta-analysis on short-term (day 7) postoperative pain showed pulpotomy to provide effective pain relief. However, because only one study provided clinical and radiographic outcome data, it was not possible to perform meta-analysis for this critical outcome [19]. That study also used a type of HCSC that is not commercially available in Europe and the findings may not be applicable to other materials. Accepting the limitations of the two included studies, the authors concluded that full pulpotomy was as successful as root canal treatment for teeth with IRP [19]. A recent preliminary trial showed that full pulpotomy was as effective as root canal treatment in terms of clinical outcome measures and quality of life outcomes but had better patient satisfaction outcome [20].

The emerging interest in vital pulp treatment (VPT) prompted the ESE [11] and the American Association of Endodontists (AAE) [21] to develop position statements and guidance on the management of deep caries and exposed pulp, which recommended adoption and promotion of VPT, and strategies aimed at preserving all or part of the pulp. However, both statements acknowledged gaps in current knowledge and the need for well-designed and adequately powered randomised trials to provide the evidence needed to change clinical practice [11, 21].

Planning and conducting adequately powered, well-designed multicentre randomised trials present huge financial and logistic challenges, and trials have limited external validity if carried out in only one centre. To overcome these limitations, prospective meta-analysis (PMA) has been suggested as an evidence synthesis method that offers many advantages including efficient adaptive design, reduction of research waste and bias, and increased generalisability and collaboration opportunities [22].

PMA is usually selected for research questions that are of high priority, often in areas related to emerging treatment strategies. PMAs that combine individual participant data (IPD) provide the highest level of evidence because of their improved statistical power and avoidance of selection and selective outcome reporting bias [23]. A key feature is that eligible studies are identified, and hypotheses and analysis strategies are specified before the results of the included studies are known [22]. Ideally, research collaboration and discussions on study questions, outcomes, and procedures should occur before initiation of any study procedures. In this project, a consortium of researchers with a proven track record of research in VPT have formulated the research question and designed the independent randomised trials that will take place in different geographical locations (multicentre), applying a standard protocol to provide data that will be pooled in a prospective meta-analysis. This approach demands that the overarching study is designed and conducted according to a protocol developed and agreed upon by the consortium. This reduces heterogeneity in case selection, operative procedures, and outcome measures. It is anticipated that adopting this international collaborative approach will address the limitations of current studies, including small sample size, heterogeneity, and their lack of generalisability and pragmatism.

### Priority research question

In mature permanent teeth with symptomatic irreversible pulpitis (P), is full pulpotomy (I) non-inferior to conventional root canal treatment (C) in terms of clinical outcomes and cost-effectiveness (O)?

To address the gap in the knowledge and provide high-quality evidence to answer the above research question, the study was set to address the following objectives:

1. To undertake a series of individual randomised trials comparing the clinical and cost-effectiveness of full pulpotomy and root canal treatment for mature permanent posterior teeth with signs and symptoms indicative of irreversible pulpitis using a standardised protocol.
2. To perform prospective meta-analysis for primary and secondary clinical outcomes data outlined below.
3. To determine the cost-effectiveness of the procedure across different jurisdictions and various healthcare delivery systems based on the primary outcome.
4. To perform a process evaluation to test the fidelity and acceptability of the intervention by patients and dentists across different cultures and healthcare delivery systems.

The *primary outcome* is a composite measure at 12 months defined as (1) the absence of pain indicative of IRP, (2) the absence of signs and symptoms indicative of acute or chronic apical periodontitis, and (3) the absence of radiographic evidence of failure including radiolucency or resorption. Failure of any part of this composite measure will equate to overall treatment failure.

The *secondary outcomes* are as follows:

- Clinical: (1) pain at days 3 and 7 post-treatment, (2) integrity of the tooth/restoration, (3) need for further interventions, (4) radiographic evidence of calcifications, and (5) positive sensibility response on electric pulp testing for the VPT arm. Outcomes 2 to 5 will be assessed at 12 months.
- Non-clinical: (6) cost-effectiveness of the treatment and (7) process evaluation (to assess the acceptability of the intervention to dentists and patients and to explore barriers and enablers to implementation).

## Methods

### Protocol design

This study is a prospective meta-analysis of individual participant data from randomised trials. Investigators currently leading vital pulp treatment studies were identified from trial registries and through personal communications and approached for contribution to the proposed PMA. The prospective meta-analysis is registered a priori in PROSPERO, registration number CRD42023446809. The protocol is written according to the PRISMA-P guidelines [24], and the review will be reported according to the PRISMA-IPD guidelines [25].

### Study eligibility criteria

All trials to be included in the PMA should be conducted in line with an established protocol, including the measurement of unified core outcomes. https://www.isrctn.com/ISRCTN49302282. The trials should be conducted by researchers with previous experience in VPT in settings of high need to ensure acceptable recruitment rates. All investigators will agree to share anonymised individual participant data for all patients who are randomised.

### Sources

The project lead and co-lead performed search with no restriction on date or language on trial registries (ClinicalTrials.gov and ICTR at https://trialsearch.who.int) and databases MEDLINE, The Cochrane Library and Web of Science using the terms, “vital pulp treatment”, “pulpotomy”, and “root canal treatment” to identify trials on vital pulp treatment. Researchers are also identified through personal communications with experts in the field.

### Participating studies/sites

Ten sites contribute to the study. These are the British University in *Egypt*; Manipal College of Dental Sciences, *India*; Jordan University of Science and Technology, *Jordan*; Khartoum University, *Sudan*; Universiti Malaya, *Malaysia*; Chiang Mai University, *Thailand*; Piracicaba Dental School, *Brazil*; Queen’s University, Belfast, *Northern Ireland*; Birmingham, Alabama, *USA*; and National University of Singapore, *Singapore*. Sites were recruited based on experience running randomised trials and performing VPT. It is anticipated that each site will randomise 50 to 60 patients between root canal treatment and pulpotomy and provide data on the primary and secondary outcomes outlined above.

### Patient eligibility criteria

The included clinical trials will adopt the following criteria:

- *Inclusion*: Patients aged 12 years or older (with a mature permanent tooth demonstrating radiographic evidence of deep caries/restorations and signs/symptoms indicative of IRP (moderate to severe spontaneous lingering pain)). Tooth responsive to cold and EPT sensibility testing, restorable, and can be adequately isolated during treatment. Only one posterior tooth (molar or premolar) per patient.
- *Exclusion:* Teeth with active periodontal disease (pocket depth > 5 mm); teeth indicated for elective root canal treatment for restorative purposes; teeth with apical periodontitis; patients with complex medical histories that may affect their caries’ experience and healing ability (immunocompromised, radiotherapy); patients who are unable to consent; history of previous trauma to the tooth; the presence of apical radiolucency; and patients who are pregnant or breastfeeding. Intraoperatively, patients with any evidence of purulence or excessive bleeding that cannot be controlled with a cotton pellet with 2–4% hypochlorite for 10 min will be excluded.

### Patient enrollment

Patients attending with symptoms suggestive of IRP will be screened for eligibility. Diagnosis of IRP is suggested if the patient presents with sharp and lingering pain triggered by thermal stimulus [often 30 s or longer after stimulus removal] and the presence of spontaneous pain. A sensibility test with a −50 °C thermal test (e.g. Endofrost) or −26 °C Endo Ice (Coltene, Cayahoga Falls, OH, USA) will be performed and periapical and bitewing radiographs clearly showing the depth of caries lesion/ restoration and periapical area should be obtained as part of the diagnostic procedure. Following assessment (both clinical and radiographic) and diagnosis, patients who fulfil the inclusion criteria and agree to participate will be randomised to one of the trial treatments.

### Randomisation

Following confirmation of the clinical diagnosis of IRP and after obtaining informed consent, participants will be randomised to receive one of two treatments: root canal treatment or complete/full pulpotomy. The unit of randomisation will be the participant. Randomisation will be completed using a block randomisation system. Randomly permuted blocks of four and eight will be generated using the computer-generated online tool Sealed Envelope™ (https://www.sealedenvelope.com/) with no stratification. Operators will be blinded to the randomisation, which will be provided in sealed envelopes for concealed allocation.

### Recruitment and retention

To ensure adequate pooled recruitment and to account for the potential of lower outcomes from some sites, we decided on an average recruitment rate of 5 patients per month per centre over a recruitment period of 12 months. Recruitment rates will be monitored by the study team on a monthly basis to ensure there is adequate recruitment. Should there be any threat to overall recruitment targets, the research team will consider the possibility of opening further sites. As it is likely that the study population will include some irregular dental attenders, the research team will also explore the use of text message reminders particularly at the 12-month follow-up stage.

### Clinical procedure

#### Root canal treatment (control)

As these are pragmatic trials, this procedure will be carried out using the methods and materials that participating clinicians would normally use for performing root canal treatment. A contemporary root canal treatment protocol in line with standards outlined in the AAE Treatments Standards [26] is expected. The procedure can be carried out in single or two visits. However, wide variations in root canal treatment protocols would make it difficult to compare with pulpotomy, so the aim is to standardise the protocols for the following variables: adequate anaesthesia, use of rubber dam, irrigation protocol with 2–4% sodium hypochlorite, working length with combined radiographs and apex locators, automated instrumentation to accompany hand instrumentation and preparation to apical size 2–3 larger than IAF, canal to be medicated with non-setting calcium hydroxide if done in two visits and root canal filling with gutta-percha and traditional sealers (warm or cold lateral condensation) and good coronal seal.

#### Full pulpotomy (experimental intervention)

The clinical procedure will be completed in one visit, but the final restoration may be placed in a follow-up visit. Following adequate anaesthesia and isolation with a rubber dam, access to the pulp will be gained following caries removal to de-roof the pulp chamber and excision of the entire coronal pulp. The pulp chamber will be irrigated with 2–4% sodium hypochlorite solution and resultant bleeding from the remaining pulp will be controlled with a cotton pellet soaked in 2–4% sodium hypochlorite solution for up to 10 min. Following complete haemostasis, the pulp stump will be covered with Biodentine (Septodont Ltd., Saint-Maur-des-Fossés, France) and the tooth permanently restored with restoration if treatment is completed in a single visit or temporised with glass ionomer cement for the final restoration to be placed in a second visit if the operator opted for 2-visit treatment.

If haemostasis at the pulp wound is not achieved within 10 min, pulpectomy and root canal treatment should be carried out and the patient will be excluded from the trial. Similarly, if no bleeding is evident when the pulp is exposed, it will be assumed non-vital, and the tooth will be excluded from the study. Every excluded patient after the randomisation will be included in the analysis as failure in the arm they are randomised to. A post-operative periapical radiograph (using a parallel cone technique) should be taken of the restored tooth following completion of treatment.

### Follow-up and data collection

#### Primary outcome data

The composite primary outcome will be assessed at a minimum of 12-month follow-up. Data to be collected include:

1. *Clinical data*: absence of pain, tenderness to palpation and percussion, presence of swelling, presence of sinus tract, pathological mobility and/or loss of responsiveness to sensibility testing (cold and/or electric pulp testing). Patient history taking and clinical examination for symptoms and clinical signs of infection such as swelling and sinus tract will be performed by a dental practitioner who is blinded to the patient’s allocated treatment.
2. *Radiographic data:* the presence of periapical radiolucency, the presence of inter-radicular radiolucency, the presence of resorption, and the presence of calcifications. An independent assessor at each site will assess the radiograph obtained at the 12-month follow-up.

#### Secondary outcome data

Secondary outcomes will be assessed as follows:

1. Postoperative pain will be recorded by patients on days 3 and 7, using a numeric rating scale (NRS). NRS is an 11-point numeric scale with 0 representing “no pain” and 10 representing “pain as bad as you can imagine”. Patients will be instructed on how to use the NRS-11 at home on days 3 and 7 after the procedure. Patients will be contacted by phone call or text message to collect their responses.
2. Structural integrity of the tooth will be assessed by a dental practitioner who is blinded to the allocated treatment at the 12-month visit using World Dental Federation (FDI) criteria [27].
3. Evidence of further interventions and adverse events will be obtained from patient records at the 12-month review visit.
4. Data for the cost-effective analysis and process evaluation will be collected as described below.

### Covariates

Data on variables that may influence the outcome of the treatment will be collected including:

1. Preoperative factors: age, gender, tooth type (molar/ premolar), cavity type (occlusal proximal)
2. Intraoperative factors: bleeding time, single vs two visit treatment, operator (endodontist, postgraduate student or general dental practitioner)
3. Postoperative factors: interval to the placement of the final definitive restoration

### Data provision and management

Outcomes and covariates data will be obtained for each participant included in each trial. Anonymised data will be entered in encrypted Excel sheets and sent to the PMA secretariat in Queen’s University Belfast via a secure server for analysis. Before analysis, data will be checked for quality and completeness by two independent assessors. Disagreement will be resolved by consensus. A data management plan will be developed in line with the data protection and handling rules of Queen’s University Belfast.

### Meta-analysis

Anonymised individual participant data from each participant in each trial will be collated, harmonised, and analysed. Heterogeneity and inconsistency are expected to be minimal due to the harmonisation arising from the standardisation of practices and outcome measures. A two-stage data analysis will be used. The first stage involves a standard regression analysis of each individual trial to produce aggregate data, including treatment effect estimates and their variances. This will be followed by inverse variance weighted meta-analysis to combine these aggregate data and produce summary statistics and forest plots. A common-effect and a random-effects model will be used. Sensitivity analysis and meta-regression will be carried out to investigate the effects of covariates: pre-operative, intra-operative, and post-operative factors, as outlined above.

Subgroup analysis will be carried out based on patient age, preoperative pain, and type of operator. Meta-analysis will be performed using Review Manager, Version 5.3 (The Nordic Cochrane Centre, the Cochrane Collaboration, Copenhagen, Denmark).

#### Missing outcome data and sensitivity analyses

The aim is to minimise the amount of missing data, supported by the limited additional visits required by the participants and collection of data from standard dental records. We had also inflated the sample size by 5% to account for possible missing data. In addition, a maximum-likelihood multiple imputation approach will be used for the management of missing data with a sensitivity comparison with the complete case dataset. Upon commencement of the procedure, some participants randomised to receive pulpotomy may require root canal treatment, in a manner that could not be established prior to treatment starting. These participants will remain in the analysis set for the primary analysis but we will exclude these participants for a subsequent sensitivity analysis.

#### Power calculation for the meta-analysis

As this is a PMA, the power of the meta-analysis will be determined after data collection is completed. To determine what would be an adequate total number of participants, we estimated the sample size for a non-inferiority trial with a binary outcome assuming no difference between the success rate of the pulpotomy and root canal treatment (90%) with a non-inferiority margin of 10%. For such a trial, a total of 380 patients randomised on 1:1 basis would have 90% power to detect a non-inferiority margin of 10%, at a 5% significance level. To account for the 25% dropout rate and 5% potential data loss/incomplete data for the 12-month outcome, 495 patients would be needed.

### Risk of bias assessment and GRADE recommendation

As individual participant data will be provided after completion of the study prior to publication of individual study reports, the risk of bias will be assessed of the study protocol and interviews of the principal investigator in each site using the original Cochrane RoB assessment tool [28] by two independent reviewers. The studies will be categorised as having “Low”, “Some concerns”, or “High” risk of bias based on an assessment of the presence of bias due to randomisation, deviations from intended interventions, missing outcome data, measurement of the outcome, selection of the reported result, and overall quality. The certainty of the evidence will be assessed with the Grading of Recommendations Assessment, Development and Evaluation (GRADE) approach [29].

### Health economic evaluation

An incremental cost-effectiveness analysis will be undertaken from the perspective of a publicly funded third-party payer. Incremental costs will be related to incremental success on the composite outcome as an incremental cost-effectiveness ratio in the main health economic analysis. The relative value for money of the interventions will be established by comparing the estimated incremental cost-effectiveness ratio (ICER) against a range of hypothetical willingness to pay thresholds informed by the literature and data collected from participants with uncertainty around the threshold level explored using cost-effectiveness acceptability curves. Sub-group analyses will be used to explore differences that may exist between groups differentiated by healthcare systems. A range of sensitivity analyses will be undertaken to examine issues such as parameter uncertainty. Data on the cost of consumables, the time taken by the dentist for each therapy, whether they are assisted and what the dentist and assistants are paid, or how much they receive from the public system for doing this work will be collected from all participating sites. Data will be collected in predesigned and validated forms. Data will be analysed initially for individual trials and then for all trials data combined using both a parametric and non-parametric approach. In the former, each observation will be weighed and a seemingly unrelated regression analysis where group membership is one of the independent variables will be undertaken. We will bootstrap the data adjusting for weights to perform non-parametric analysis.

### Process evaluation

A process evaluation will be undertaken alongside each trial to study acceptability for dentists and patients and treatment fidelity for the dentists. This will aim to capture the contextual factors that shape the intervention’s implementation. The method will involve focus group meetings and structured interviews to collect the required data. Predestined treatment acceptability questionnaires will be used, and interviews will be conducted using an agreed matrix.

### Ethical approval

Based on the ethics guidance issued by the Medical Research Council (UK), this study did not require separate ethics committee approval for the following reasons: (1) investigators of each of the original studies obtained local ethics committee approval and written, informed patient consent (and assent as needed) will be obtained from all participants in the trials included in the PMA. (2) The project uses anonymised data from individuals recruited to the original studies who cannot be identified in the PMA.

### Project management

**Fig. 1:**
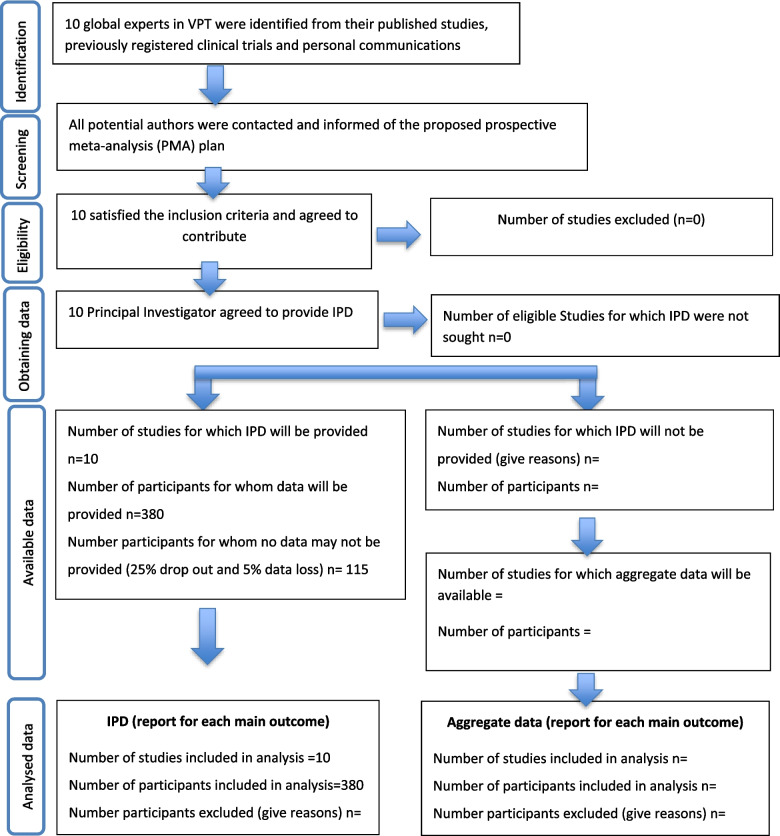
Study flow diagram

The project management structure consists of the following: (1) an international steering committee comprising principal investigators of the included sites, who are all expert Endodontists with interest in VPT. The steering committee will meet every 3 months to monitor the conduct of the trial; (2) Research and Data Management Committee which develop the methods and ensured the attainment of project milestones (Fig. 1). This includes the project lead (IEK), co-lead (HD), trial methodologist (MC), and health economist (CO’N). Only these individuals had access to the data obtained from individual studies. As this is, a low-risk trial, data monitoring committee is not required.

### Protocol amendments

If amendments are required, approval will be requested from the relevant ethics Committee who will advise whether these are minor or substantial. If substantial amendments are made to the protocol, the trial sponsor in each site will be informed. The PI will notify the centres and a copy of the revised protocol will be sent to the PI to add to the Investigator Site File. The amendment will be marked as a new version of the protocol and clearly described as amended, with the date of the amendment. The protocol in the clinical trial registry will also be updated. Any deviations from the protocol will be fully documented using a breach report form.

### Post-trial care

There is no anticipated harm and compensation for trial participation. In case of failure of any of the treatments provided, the participant will be offered appropriate care in line with clinical practice.

### Patient and public involvement (PPI)

Patient and public involvement contributed to the protocol development when initially incepted in N. Ireland with input from “The Public Involvement Enhancing Research Northern Ireland (PIER-NI)”. The trials are coordinated by the steering committee which monitor trial progress with support from the local chief and co-investigators via emails and online meetings. “The steering committee will meet every 3 months to monitor the conduct of the trials”.

## Discussion

The aim of this prospective meta-analysis is to pool individual participant data from a linked series of individual randomised trials investigating the clinical and cost-effectiveness of pulpotomy compared with conventional root canal treatment for teeth with signs and symptoms indicative of IRP in order to provide estimates of treatment effect. Although the aims of the study might be met by running a single, multicentre randomised trial, the PMA approach offers additional benefits to the advantages of traditional multicentre trials. These include the flexibility for each study to answer additional local questions, efficiency, and generalisability [30]. Unlike single multicentre randomised trials, the decentralised nature of the PMA reduces the impact of issues such as the need for every site to meet the requirements of the Institutional Review Board in each of the other sites and other approval processes, which often delay the start of the trial and increase associated costs.

Our approach to standardise key element of the trial protocol and agree on the core outcome will reduce heterogeneity and increase the power and precision of the PMA. This will also reduce the need for extensive harmonisation that is often required in IPD meta-analysis. On the other hand, the pragmatic nature of the trials will allow for flexibility whereby each site can choose a local protocol (for example, single or two-visit treatment) and use of permanent restoration (resin-based composite vs amalgam). Such pragmatism is advantageous and should provide “real world” scenarios for treatment that will be more generalisable to dental practice around the world. In addition, it should provide opportunities to test if these variables influence treatment outcomes.

One of the main advantages of PMA is the opportunity for collaboration and data sharing, which are essential for the transparent reporting of research findings. The development of the collaborative consortium for this study provided an opportunity for networking, mentorship, and sharing of expertise, which will help to build dental research capacity internationally. The establishment of the collaborative consortium has also demonstrated the feasibility of running global VPT studies and should facilitate further studies that are needed to answer dental research questions of global relevance.

## Conclusion

PMA is an efficient and rigorous methodology to answer emerging research questions. In view of the limited evidence on the clinical and cost-effectiveness of pulpotomy compared with conventional treatment (root canal treatment), this research project study will contribute information for evidence-based decision-making related to the management of teeth with signs and symptoms indicative of IRP.

## Trial status

PMA Protocol V1 was registered in PROSPERO on 08 February 2023, registration number CRD42023446809. The anticipated PMA start date is 10 February 2023, and the completion date is 03 February 2026. Trials included in the PMA are available at:

https://clinicaltrials.gov/ct2/show/NCT05726357

https://clinicaltrials.gov/ct2/show/NCT04922229

https://clinicaltrials.gov/ct2/show/NCT05279781

https://www.isrctn.com/ISRCTN49302282

https://ctri.nic.in/Clinicaltrials/login.php: CTRI/2023/03/051186

https://www.thaiclinicaltrials.org/: TCTR20230526002

https://clinicaltrials.gov/study/NCT05964933?term=NCT05964933&rank=1

### Supplementary Information

**Additional file 1. **PRISMA-P 2015 Checklist.

## Data Availability

Not applicable.

## Abbreviations

AAE: American Association of Endodontists
ESE: European Society of Endodontology
GRADE: Grading of Recommendations Assessment, Development and Evaluation
IDP: Individual participant data
IRP: Irreversible pulpitis
PMA: Prospective meta-analysis
UK: United Kingdom
VPT: Vital pulp treatment
